# Strategic planning for bioanalytical automation: managing growth successfully

**DOI:** 10.1155/S1463924692000117

**Published:** 1992

**Authors:** Julie J. Tomlinson

**Affiliations:** Department of Clinical Pharmacology Glaxo Inc. Five Moore Drive Research Triangle Park North Carolina USA

## Abstract

Bioanalytical automation expanded at Glaxo Inc. from 1987 to 1991 by cycling through periods of justification, planning, implementation, obstacle-jumping and success, which justified continued cycling. In 1990 it became evident that the technology and its growth needed to be planned and the resources had to be managed. A Strategic Plan was researched and prepared. The plan describes the mission, values, goals and structure of the Bioanalytical Automation Group and the most important requirements for achieving those planned goals, including: (1) Long-term management commitment; (2) Trained, dedicated personnel; (3) Quality facilities; (4) Teamwork; and (5) Investment in automationcompatible equipment. The strategic plan has been in effect for over a year; current status, history, and the future are discussed in this article.


Journal of Automatic Chemistry, Vol. 14, No. 2 (March–April 1992), pp. 47–50

### Strategic planning for bioanalytical automation: managing growth successfully

**Julie J. Tomlinson**
*Department of Clinical Pharmacology, Glaxo Inc., Five Moore Drive, Research Triangle Park, North Carolina, USA*

*Bioanalytical automation expanded at Glaxo Inc. from 1987 to 1991 by cycling through periods of justification, planning, implementation, obstacle-jumping and success, which justified continued cycling. In 1990 it became evident that the technology and its growth needed to be planned and the resources had to be managed. A Strategic Plan was researched and prepared. The plan describes the mission, values, goals and structure of the Bioanalytical Automation Group and the most important requirements for achieving those planned goals, including: (1) Long-term management commitment; (2) Trained, dedicated personnel; (3) Quality facilities; (4) Teamwork; and (5) Investment in automation-compatible equipment. The strategic plan has been in effect for over a year; current status, history, and the future are discussed in this article.*

### Introduction

The Bioanalytical Robotics laboratory at Glaxo Inc. has continually progressed through a series of justification, planning, implementation, obstacle-jumping and success during its development. Within those cycles there have been many smaller cycles to justify purchases, headcount, workload and deadlines, but the five major cycles have been the driving force in terms of ability to grow, be successful and provide the senior management commitment needed to negotiate resources. The laboratory is currently in the fifth major cycle of growth.

These major cycles are described here in detail, leading up to the formal strategic plan that is now used to pace growth and to adjust to unforeseen obstacles and needs.

### The present

Glaxo Inc. is the US member of the UK-based multinational company, Glaxo Holdings. There are 41 000 employees in its 70 world-wide companies. The US operation is the second largest Glaxo company, with 4600 staff. Worldwide, Glaxo has at least 16 full laboratory robot systems; 12 of those systems are at Glaxo Inc. in North Carolina.

Glaxo Inc. has three laboratory robotics groups, including (1) a group in QA/QC with four fully automated robot systems and a variety of different types of workstations; (2) a group in Analytical Chemistry, with three robotic systems; and (3) the Bioanalytical Robotics group in the Clinical Pharmacokinetics Department, the focus of this paper.

The Clinical Pharmacology Department currently comprises two sections: Pharmacokinetics and Bioanalytical. The Pharmacokinetics Section is the primary customer of the Bioanalytical Section; other customers of Bioanalytical include Medical, Pharmaceutics, Toxicology and Glaxo UK. Most of Bioanalytical's work provides service to the Pharmacokinetics Section – drug project teams are formed with this section to work on clinical projects supporting Phases I to IV of drug development.

The Bioanalytical Section of Clinical Pharmacology comprises four subgroups: Biopharmaceutics, Method Development, Contract Analytical and Robotics, each with its own manager. Staff are cross-trained between the subgroups and they can rotate between them for career development. All of the groups engage in some form of method development. Contract Analytical externally completes about 30% of Bioanalytical's work, about 10–20% is completed manually in-house and the rest, over half, is done in robotics. Three years ago about 70% of Bioanalytical's work was contracted out, compared with about 30% today.

In Bioanalytical Robotics there are four fully automated robotic systems that primarily perform solid phase extraction with on-line HPLC analysis. The Group has liquid-liquid extraction and protein precipitation extraction capability, but assays are developed and validated using solid phase extraction because it is the most robot-compatible way to clean up human serum and urine. Chemists and university consultants with solid phase extraction expertise are employed to help develop quality solid phase extraction procedures so that they can be easily automated. The HPLC systems are on-line, sometimes with two HPLCs per robot to increase throughput. The robots can monitor and control their HPLC statuses and other conditions as needed.

The four Bioanalytical robots are all Zymark PyTechnology systems (Zymark Corporation, Hopkinton, Massachusetts): one purchased in 1989, solely analyses ranitidine; a Zymate XP robot works in parallel in drug development with a robot at a Glaxo UK facility; a 1990 system is used for method development and work overflow of various drugs; and the fourth, purchased in 1987, which analyses five different drugs, is used for method development using experimental design software and will soon be fitted with equipment and software to function as an Expert System with HPLC/solid phase extraction method design and development capability.

There are three full-time scientists and one manager working in Bioanalytical Robotics, plus rotational scientists from the other Bioanalytical groups and scientists from Glaxo UK who come to train in the technology.

© Zymark Corporation 1992

The scientists and the four robots currently analyse 20–30 000 samples per year, limited primarily by workload and secondarily by headcount.

Five robotic assays and two assays in automation development have been validated. The robots undergo complete assay validation and cross-validation under the same SOP criteria as manual assay validation and analytical equipment validation. The justification for the development and implementation of a robotic assay is determined by project deadline priority, with FDA response being the highest priority, followed by NDA submissions; other prioritization schedules are negotiated with customers.

### The past

Automation began in 1986 as a strategic investment, as evident by the justifications that management gives for bringing automation experience into the department and allowing time for its development:

(1) Bioanalytical is a local application for automation.

(2) Many of the serious bugs in the technology had been worked out.

(3) The employees were getting burned out.

(4) It would be interesting to see what it can do for quality and/or productivity.

(5) Automation was the future and we should get started soon to be leaders in the effort.

Few plans, other than hiring the chemist and buying a robot, were made during the two years of development and few expectations were set upon implementation.

To get from there to where we are now, Bioanalytical Robotics has repeatedly gone through cycles of justification, planning, implementation, obstacle-jumping and success (see figure 1). The cycles are still going on today, but the individual cycle-times have become shorter each time more evidence is shown to senior management that automation achieves its goals.

The first cycle, lasting about two years, started in 1986 when the first automation-experienced analytical chemist was hired into Bioanalytical (see table 1). That chemist

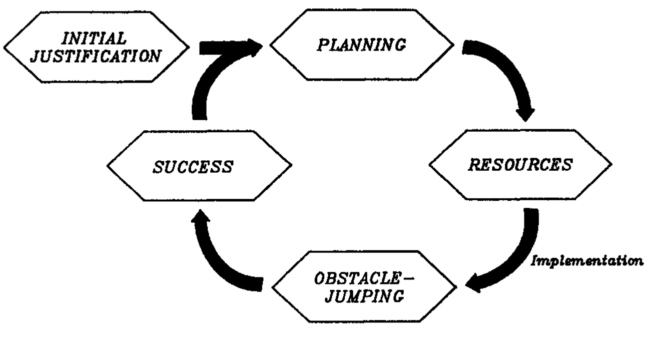

*Figure 1. Continuous growth cycle of Bioanalytical Robotics in Glaxo Inc.*

*Table 1. The first growth cycle of justification, planning and success.*

| | |
|---|---|
| Initial justifications: | The application was logical<br>The technology had improved<br>The employees were burned out<br>Quality/productivity may be improved<br>Automation was the future. |
| Initial plans: | Hire an experienced chemist<br>Contract most work out<br>Purchase a robot<br>Train the other scientists<br>Don't expect too much. |
| Obstacles: | Technology<br>Resentment<br>Fear<br>Threat. |
| Successes: | First assay developed and validated<br>Comparison with manual assay gave good results. |

was given time to develop, automate and validate an assay. The first year was spent using workstation technology and the second year using a full Zymate system. During that dedicated time more than half the laboratory's work was contracted out to allow time to develop automation without burning out the five to six chemists who then completed the work manually.

The first success came with the validation of a fully-automated assay to measure ranitidine in serum, followed by the completion of the first clinical study on a robot. That first study was analysed manually at a contract laboratory, then repeated on a robot system with no difference in the results. After that finding, the robot was regularly used for analysis of clinical study samples. A year later another study was completed, both robotically and manually, and the results were compared using statistical tests of significance. There was no difference in accuracy between the methods, and the results from samples run on the robot showed that the robotic assay is significantly more precise than the manual assay. Those results were presented at ISLAR in 1989 [1].

The biggest obstacles during that period were negative reactions from co-workers doing manual work, including resentment of the automation chemist who was 'playing with the robot', threat of loss of job security, loss of assay ownership and intimidation at the complexity of the equipment. At the earliest stages of implementation, the lack of equipment suited to bioanalytical applications was also an obstacle that was soon overcome by technological advances in the laboratory automation industry.

The successes of the first cycle justified the second cycle during which more definite plans and expectations were put into place. From the start in 1986 to today there have been five cycles. They are easiest identified by their major milestones of success. Each success helped to justify more resources, which gave way to new successes (table 2). Each successive cycle was shorter than the one before it:

*Table 2. Bioanalytical robotics growth cycles from 1987 to present.*

| | Cycle 1 1987–89 | Cycle 2 1989–90 | Cycle 3 1Q90–3Q90 | Cycle 4 3Q90–1Q91 | Cycle 5 1Q91–now |
|---|---|---|---|---|---|
| Resources | | | | | |
| No. of peoople[1] | 1 | 1 | 2 | 3(+1) | 3(+1) |
| No. of robots | 1 | 1 | 2 | 3 | 4 |
| Lab space[2] | 1 | 1 | 4 | 4 | 6 |
| Obstacles | Technology<br>Fear<br>Resentment<br>Threat | Fear<br>Resentment<br>Threat | Fear<br>Space<br>Data handling | Headcount<br>Training<br>Data handling | Headcount<br>Training<br>Data handling |
| Successes | 1st validation<br>Repeated study | Improved quality<br>1st clinical study completed | 2nd validation<br>2× throughput | International cooperation<br>Strategic Plan | 4th validation<br>5th validation<br>13 000+ samples |

1. Numbers in parentheses indicate visiting scientists.
2. Expressed in units of number of robot systems the space can accommodate.

the first was two years, the second was one year, the third was about nine months, the fourth was six months and the fifth has also been about six months. The shortening in cycle time was primarily due to the increase in senior management commitment with each success, which spawned increases in automation headcount, equipment, workload, space and the other resources essential for growth of robotics.

### Strategic planning

In mid 1990, during the third cycle, management was confident and committed enough to robotics to formally plan its future organization, functions and growth. A strategic plan was then produced for Bioanalytical Robotics. The intention was to determine and document where the group was going and what resources would be needed to get there.

First, the entire department formulated and unanimously agreed on a mission statement and a set of values for all of its groups. One of those value statements included productivity and quality, both of which had by then been exemplified by robotics. It was then decided that the best way to write a comprehensive plan for the robotics group was to conduct research by talking to people with experience of different stages of laboratory automation growth. The most valuable source of information turned out to be the 1990 ISLAR. The justifications, planning processes, obstacles and resource requirements that other users experienced during laboratory automation were compared and the information gathered formed the backbone of the stategic plan.

The research showed the following experiences of other robotics groups, regardless of their industries or applications [2,3,4,5,6]:

The justifications for (successes of) implementing automation are:

(*a*) Increased productivity, quality and efficiency.

(*b*) Safety.

(*c*) Expanded customer base.

(*d*) Increased job satisfaction/lower employee burnout.

(*e*) Greater departmental visibility.

(*f*) Updated technology.

The resources those justifications/successes required are:

(*a*) Senior management commitment.

(*b*) Appropriate laboratory facilities.

(*c*) Trained, dedicated personnel.

(*d*) Teamwork.

(*e*) Investment in compatible peripherals.

The obstacles that occurred most frequently are:

(*a*) Data collection and management limitations.

(*b*) Employee threat and intimidation.

(*c*) Space limitations.

All sources of information were emphatic that without commitment from senior management, the resources are not attainable, the successes are not achievable, and the obstacles cannot be overcome.

Another common issue that arose when speaking with people at ISLAR was that, with the growth of automation, people take the robots more seriously and anthropomorphism ceases. This is not something we have found to be true – the author's group takes robot-naming more seriously than ever and the names are used to identify the robot systems when away from them, such as in a meeting.

Also noteworthy from the information collected was that the successful robot users eventually evolved dedicated, centralized robotics groups that develop the systems to be used as turnkey instruments by staff that are inexperienced in automation. The author's group has evolved from: (1) one person who develops and uses the systems to (2) a group of people who develop and use them to (3) setting up some assays that are turnkey for inexperienced users from other groups while also running systems as end-users.

All the above information was compiled and used to write the strategic plan, which detailed the following information for one year and for three years:

(1) Missions and goals.

(2) Functions.

(3) Customers.

(4) Group organizational description.

(5) Workload management.

(6) Resources required (people, equipment, space, commitment).

(7) Potential barriers.

A more detailed outline is shown in figure 2.

*I. Departmental Mission, Values and Goals*

*II. Mission and goals of Robotics Group*
- *A. Description of Group's functions*
  - *1. Services to be performed*
  - *2. Customers, by priority*
  - *3. Goals of Group*
- *B. Description of Group*
  - *1. Where it will fit into the organization*
  - *2. How the Group will be organized*
  - *3. How the Group's workload will be managed*
- *C. Resources required to achieve the goals of Strategic Plan*
  - *1. Personnel*
  - *2. Equipment, software and systems*
  - *3. Facilities*
  - *4. Intra- and inter-departmental relationships*

*III. One-year goals of Group*
- *A. Description of Group*
- *B. Description of one-year goals*
  - *1. Steps to be taken to attain resources*
  - *2. Steps to be taken to develop customer relationships*

*IV. Potential barriers to achieving goals*
- *A. Physical barriers*
  - *1. Space*
  - *2. Personnel*
  - *3. Budget*
- *B. Political barriers*
  - *1. Reorganization*
  - *2. Commitment from staff and Senior Management*

*Figure 2. Strategic plan for Robotics Group.*

The plan has been in effect for over a year. It was reviewed for update at six months, with no changes necessary. There will probably be some adjustments to it in the future.

### The future

The group's work and growth is steady and the specific resources needed to grow according to the strategic plan have been selected.

The following technological projects will be pursued in 1992:

(1) Expert systems for intelligent control and method development.

(2) A software-, hardware, robot- and user-friendly data acquisition system.

(4) Bar-coding.

(5) Novel sample preparation techniques.

(6) RIA/ELISA.

(7) GC sample preparation.

The following political projects are being worked on:

(1) Technology transfers to and from other Glaxo groups, domestic and international.

(2) Reorganization, necessitating re-education of a new line of senior management.

(3) Quality/productivity/efficiency assessment.

(4) Customer base expansion.

### References

1. Lloyd, T. L. and Lang, J. R. Robotic vs. manual HPLC assay: precision and accuracy comparison for ranitidine in human serum. *Advances in Laboratory Automation Robotics* (Zymark Corporation, Hopkinton, Massachusetts, 1990), 73.
2. Taylor, G. L., Smith, T. R. and Kamla, G. J., Robotics fulfill a strategic need. *Advances in Laboratory Automation Robotics* (Zymark Corporation, Hopkinton, Massachusetts, 1991), 1.
3. Kushinsky, S., Managing robotics in the bioanalytical/metabolic environment of a pharmaceutical company. *Advances in Laboratory Automation Robotics* (Zymark Corporation, Hopkinton, Massachusetts, 1991), 63.
4. Elnenaey, E. A., Pluscec, J. and Fernandez, V. Challenges facing modern automated laboratories: robotic applications. *Advances in Laboratory Automation Robotics* (Zymark Corporation, Hopkinton, Massachusetts, 1991), 77.
5. Hamilton, S. D., Managing an automation development group. *Advances in Laboratory Automation Robotics* (Zymark Corporation, Hopkinton, Massachusetts, 1991), 91.
6. Plummer, G. F., Six years of robots. *Advances in Laboratory Automation Robotics* (Zymark Corporation, Hopkinton, Massachusetts, 1991), 109.

